# Supplementary material for: Increased artificial illumination delays urban autumnal foliar senescence
Source: Nat Commun. 2026 Jan 3;17:1526. doi: 10.1038/s41467-025-68246-7 (PMC12891584; doi:10.1038/s41467-025-68246-7)
Supplement: Supplementary file 1 — Supplementary Information [file 41467_2025_68246_MOESM1_ESM.pdf]

Supplementary Materials for

# Increased artificial illumination delays urban autumnal foliar senescence

Yang Chen, Wendi Qu, et al

***Corresponding author:** wucy@igsnrr.ac.cn (C.W.)

**The PDF file includes:**

Supplementary Figures 1–18

Supplementary Tables 1–2

References 1–30

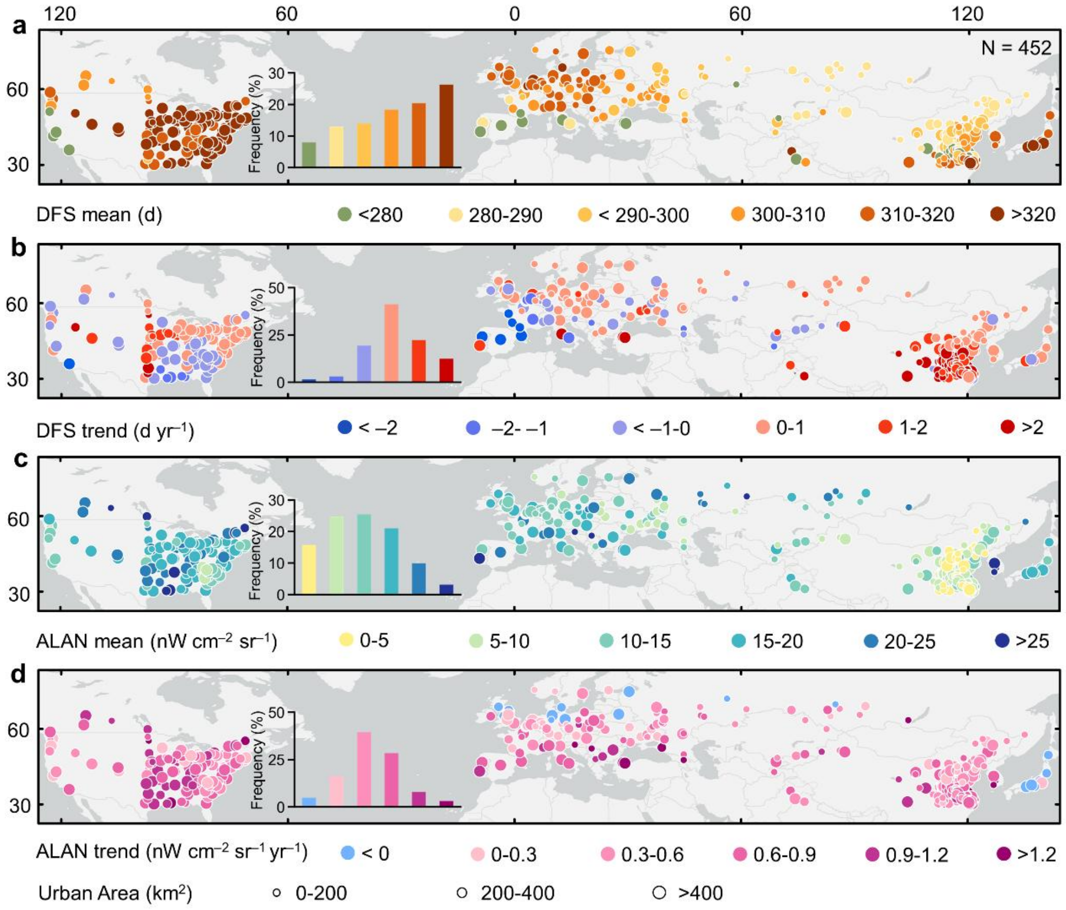


**Supplementary Figure 1.** Spatio-temporal patterns of the date of foliar senescence (DFS) and artificial light at night (ALAN) intensity from 2001 to 2020. **a**, Spatial distribution of DFS. **b**, Temporal trends in DFS. **c**, Spatial distribution of ALAN intensity. **d**, Temporal trends in ALAN intensity. N represent the number of cities.

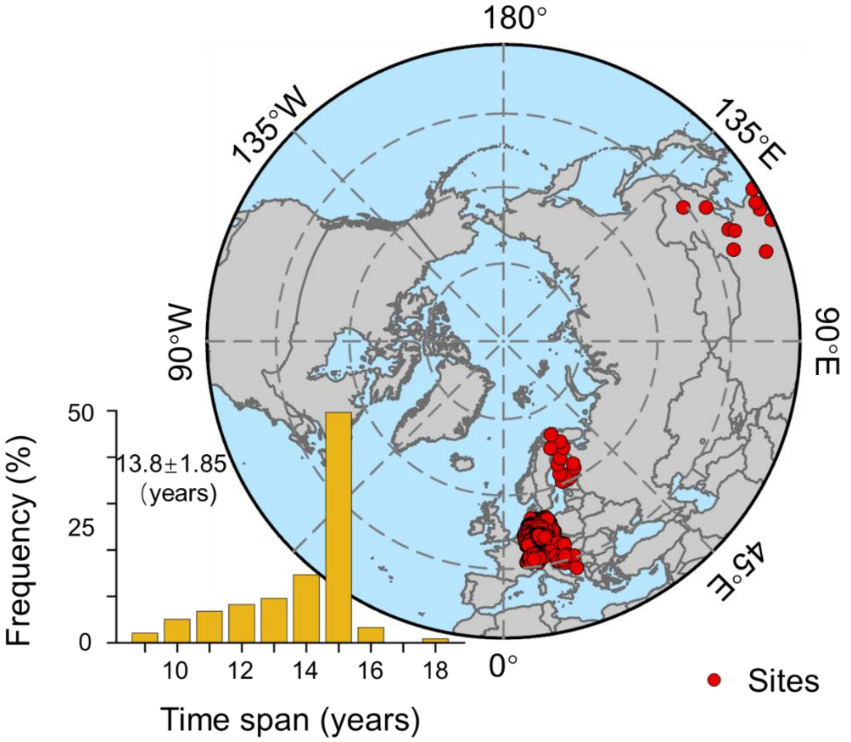


**Supplementary Figure 2.** Spatial distribution of records from the China Phenological Observation Network (CPON, n = 10) and the Pan European Phenology Project (PEP725, n = 1209), where n represents the number of sites. The subplot in the lower left corner illustrates the frequency distribution of the time span for all sites.

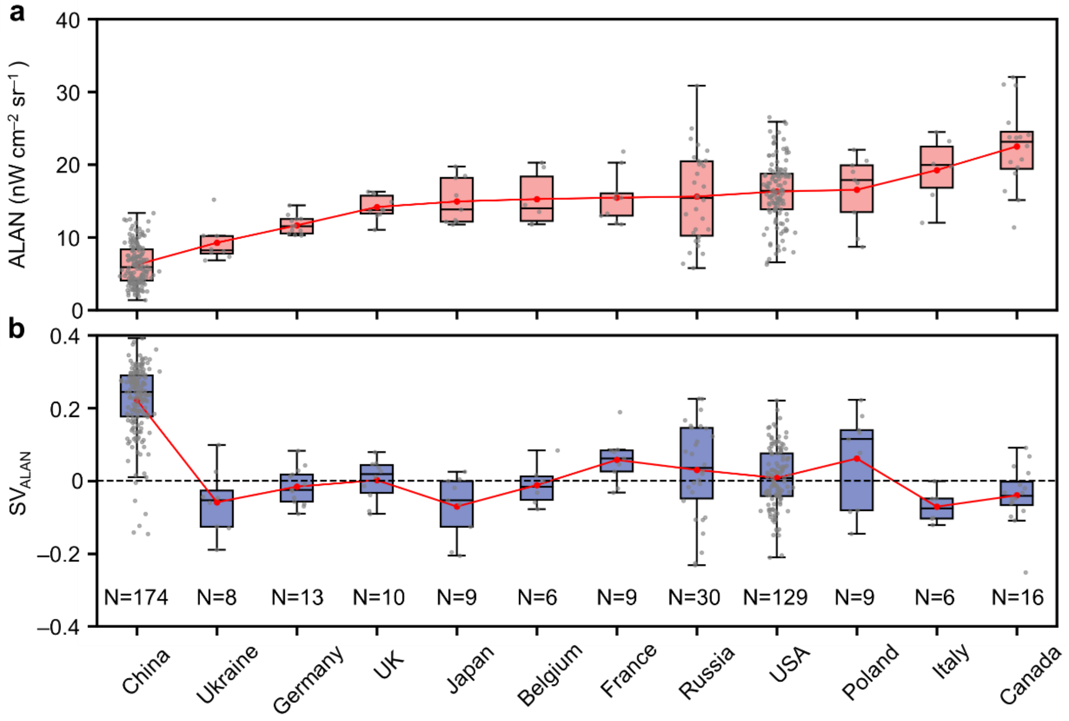


**Supplementary Figure 3.** National-scale analysis of the relationship between ALAN sensitivity ($SV_{ALAN}$) and ALAN intensity. **a**, ALAN intensity across different countries. **b**, $SV_{ALAN}$ across different countries. Box plots display medians (horizontal lines), the 25th and 75th percentiles (box edges), and minimum and maximum values (whiskers). N represent the number of cities.

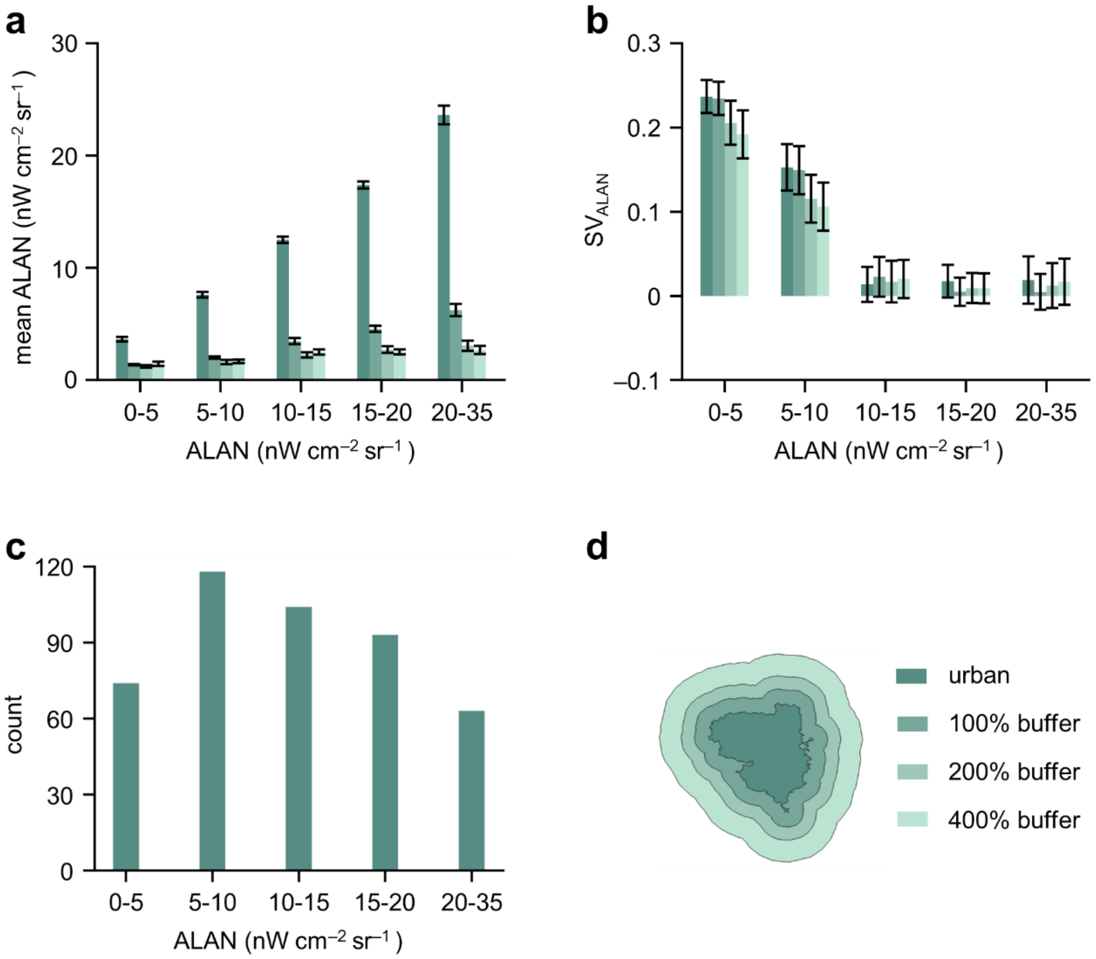


**Supplementary Figure 4.** Buffer zone scale analysis of the relationship between ALAN sensitivity ($SV_{ALAN}$) and ALAN intensity. **a**, The mean and standard deviation of light intensity for cities and their buffer zones within each ALAN intensity interval. **b**, The mean and standard deviation of ALAN sensitivity for cities and their buffer zones within each light intensity interval. Data are presented as mean values ± standard deviations for (**a**) and (**b**). **c**, The number of cities within each light intensity interval. **d**, Schematic diagram showing urban and their 100%, 200%, and 400% buffer zones.

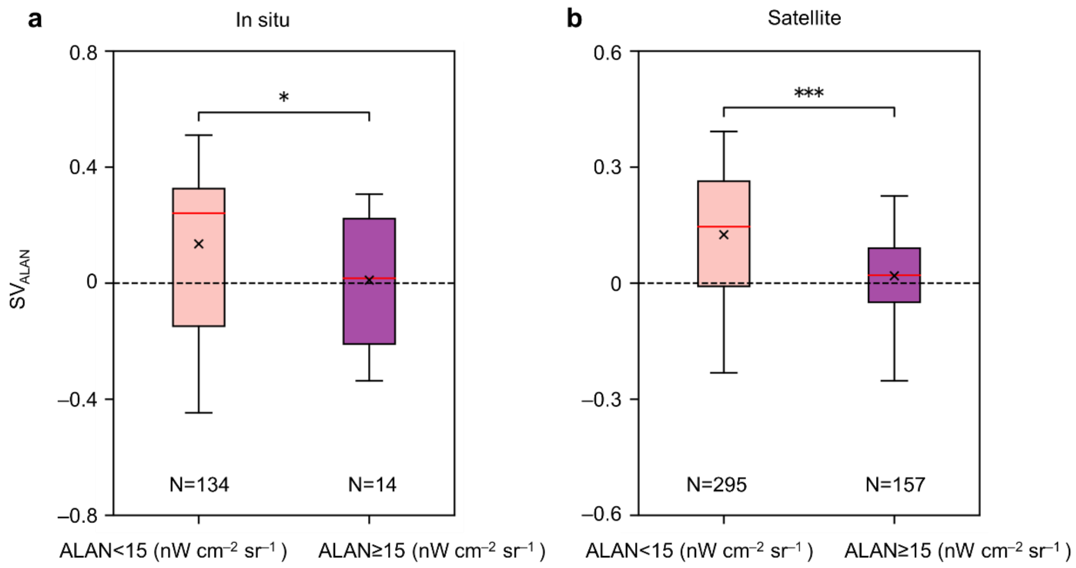


**Supplementary Figure 5**. Comparison of ALAN sensitivity ($SV_{ALAN}$) between regions with low- and high-intensity ALAN. **a**, In-situ observations analysis. **b**, Satellite remote sensing analysis. * and *** indicate significant difference between the two groups using the analysis of variance at $P < 0.05$ and $P < 0.001$, respectively. Box plots display medians (horizontal lines), the 25th and 75th percentiles (box edges), and minimum and maximum values (whiskers). N represent the number of cities.

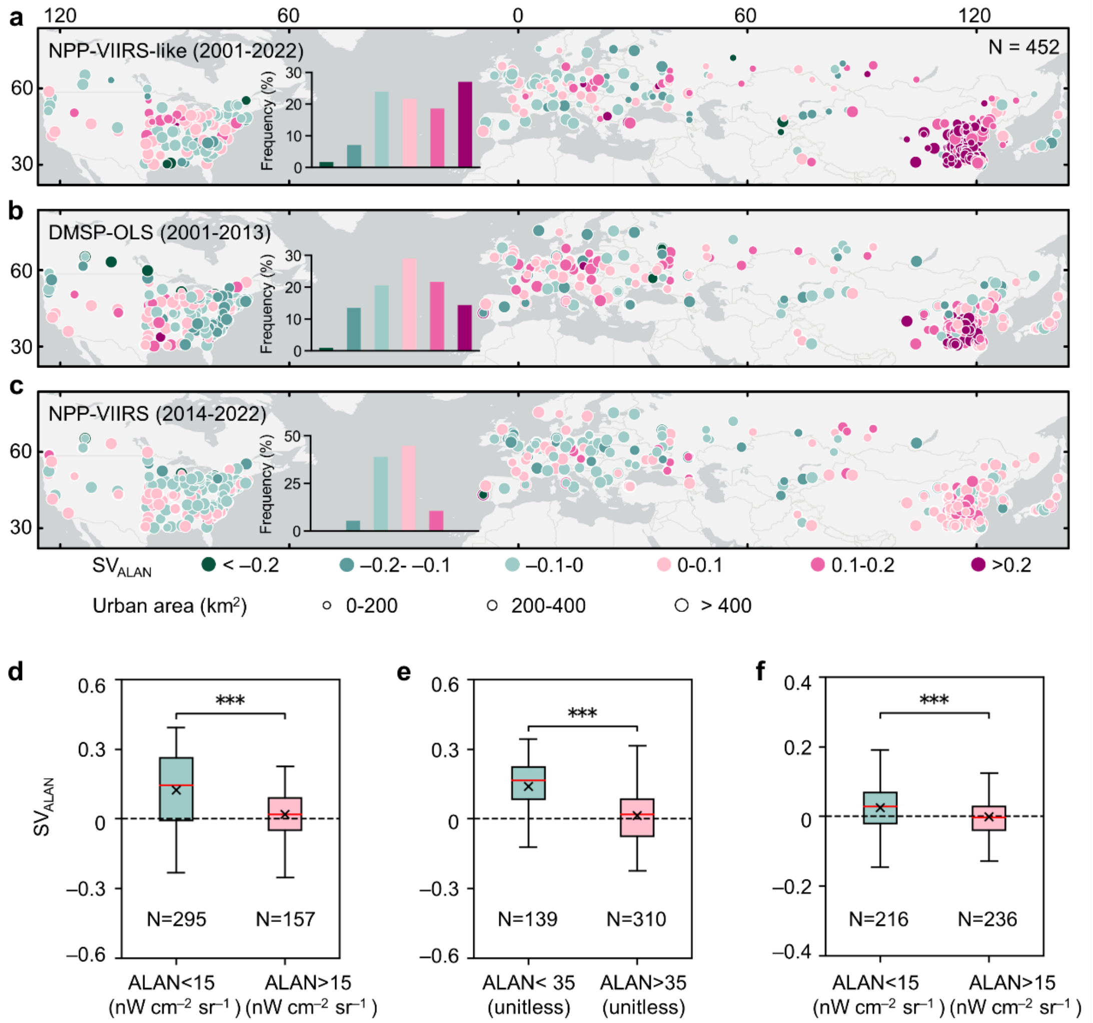


**Supplementary Figure 6.** Spatial patterns and intensity responses of the sensitivity ($SV_{ALAN}$) of the DFS to ALAN. Spatial distribution of $SV_{ALAN}$ (**a-c**) and differences in $SV_{ALAN}$ between low- and high-ALAN intensity regions(**d-f**) derived from the original and two comparative analyses. Box plots display medians (horizontal lines), the 25th and 75th percentiles (box edges), and minimum and maximum values (whiskers). N represent the number of cities.

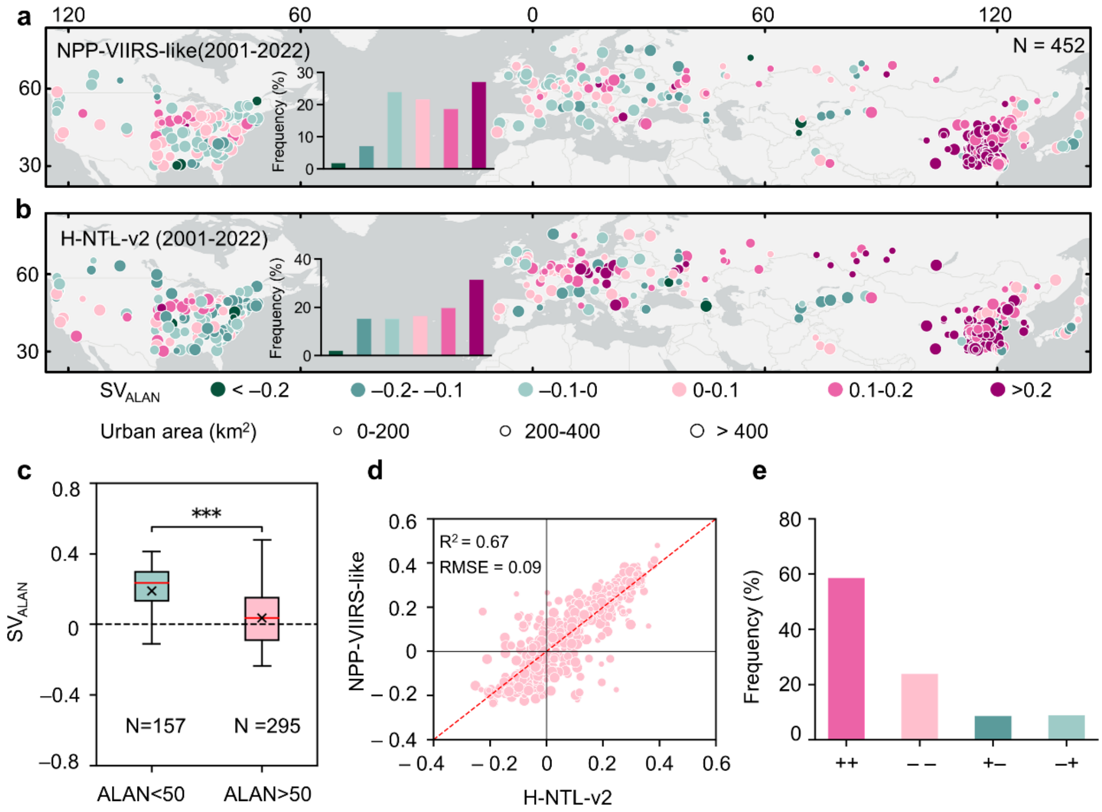


**Supplementary Figure 7.** Comparison of the sensitivity ($SV_{ALAN}$) of DFS to ALAN derived from two nighttime light datasets. Spatial patterns of $SV_{ALAN}$ estimated from the NPP-VIIRS-like (**a**) and H-NTL-v2 datasets (**b**), respectively. **c**, Differences in $SV_{ALAN}$ between low- and high-ALAN intensity regions based on the H-NTL-v2 dataset. Box plots display medians (horizontal lines), the 25th and 75th percentiles (box edges), and minimum and maximum values (whiskers). The numbers in brackets represent the number of cities(n). **d**, Linear relationship of $SV_{ALAN}$ values derived from the two datasets; the red dashed line indicates the 1:1 reference line. **e**, Frequency of matched $SV_{ALAN}$ sign patterns between the two datasets, where "++" and "—" indicate consistent positive and negative sensitivities, and "+–" and "–+" indicate opposite sensitivities. N represent the number of cities.

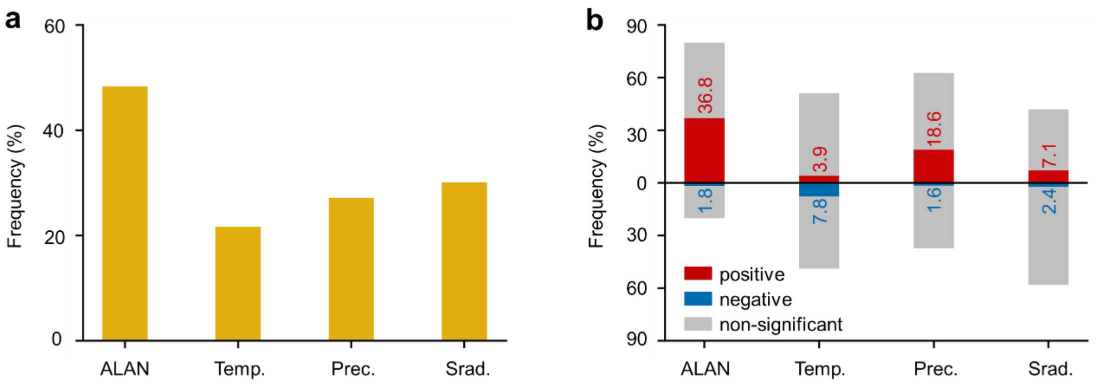


**Supplementary Figure 8.** Causality and correlation between ALAN and DFS using the Peter-Clark Momentary Conditional Independence Plus (PCMCI+) method. **a**, The frequencies of the time series with causality from climatic drivers (temperature: Temp., precipitation: Prec., shortwave radiation: Srad.) and ALAN to DFS. **b**, The distribution of the time series with significantly positive, negative, and non-significant partial correlations between climatic drivers/ALAN and DFS after excluding the other variables. Significance was set at $P < 0.1$. A two-sided $t$-test was used to assess the significance of the partial correlation analysis.

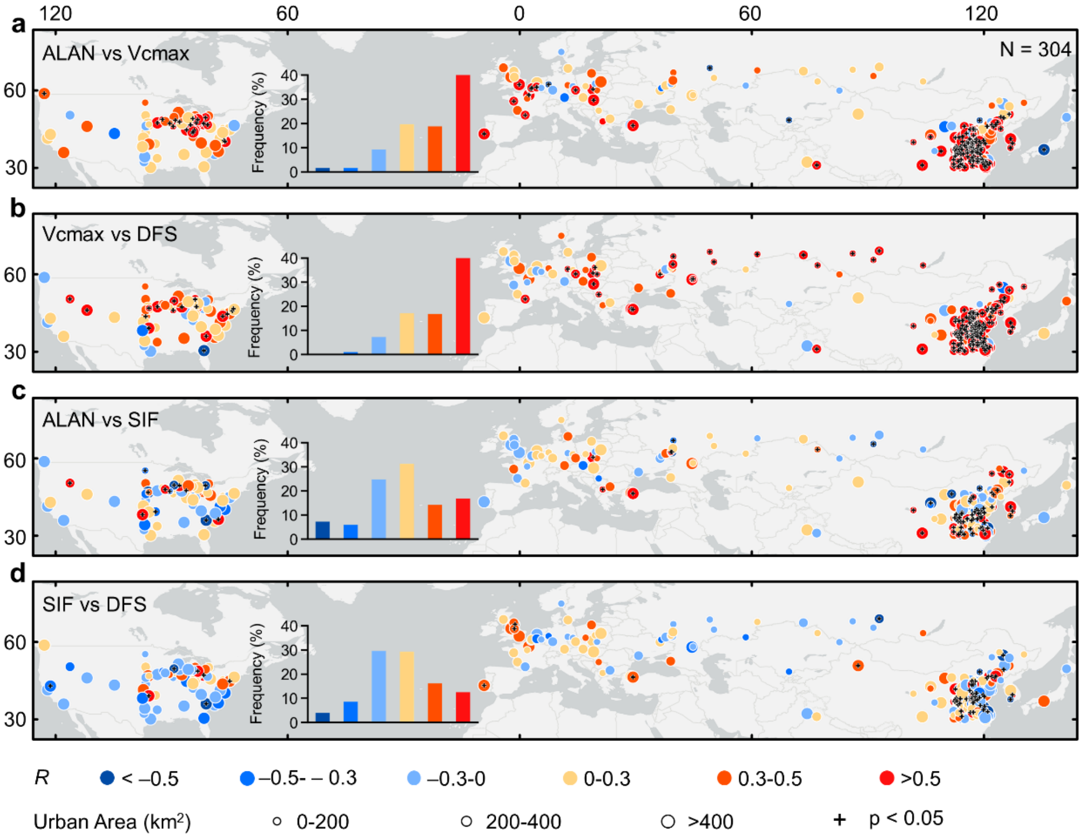


**Supplementary Figure 9.** Spatial patterns of the mediating pathways linking ALAN to DFS. Spatial pattern of partial correlations between ALAN and photosynthesis indicators, including the maximum rate of carboxylation ($V_{cmax}$) (**a**), solar-induced chlorophyll fluorescence (SIF) (**c**). Spatial pattern of partial correlations between photosynthesis indicators and DFS, including $V_{cmax}$ (**b**), SIF (**d**). + in the circle represents $P < 0.05$. A two-sided *t*-test was used to assess the significance of the partial correlation analysis. N represent the number of cities.

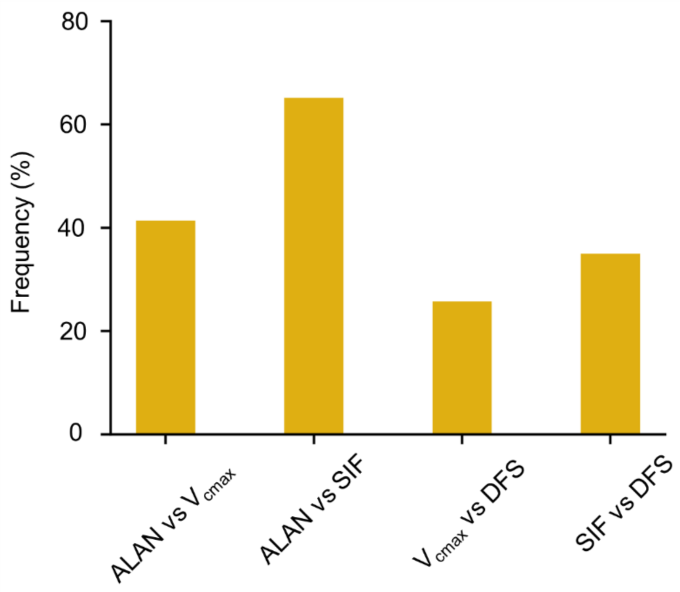


**Supplementary Figure 10**. Frequencies of causal relationships along the ALAN-photosynthesis-DFS pathway using the PCMCI+ method (Methods). Photosynthesis indicators include the maximum rate of carboxylation ($V_{cmax}$) and solar-induced chlorophyll fluorescence (SIF).

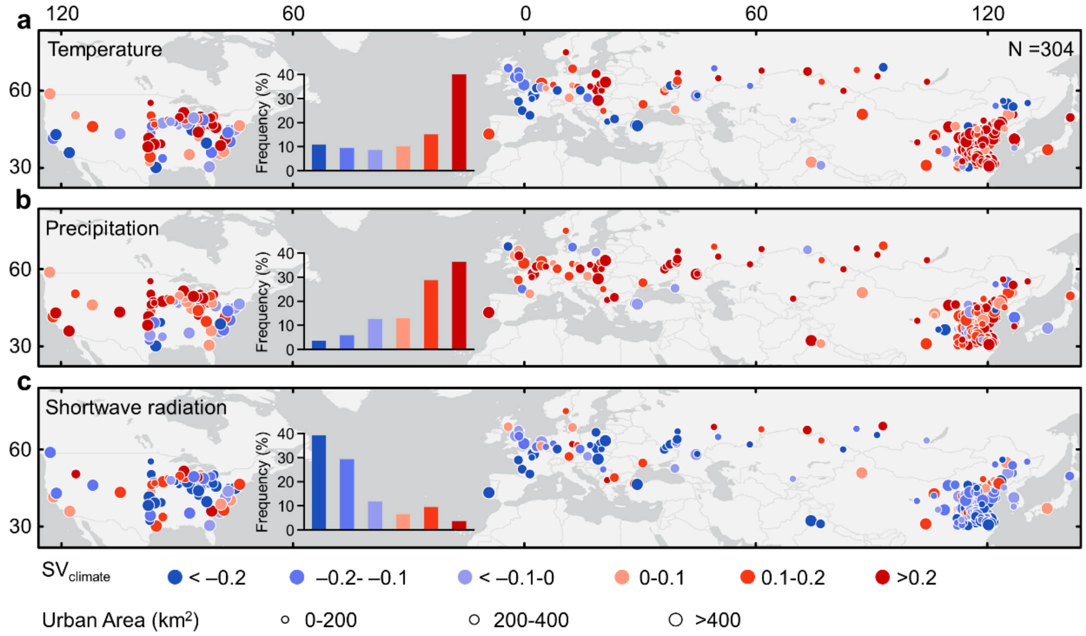


**Supplementary Figure 11.** Spatial pattern of DFS's sensitivity to climatic factors ($SV_{climate}$): temperature (**a**), precipitation (**b**), and shortwave radiation (**c**). N represent the number of cities.

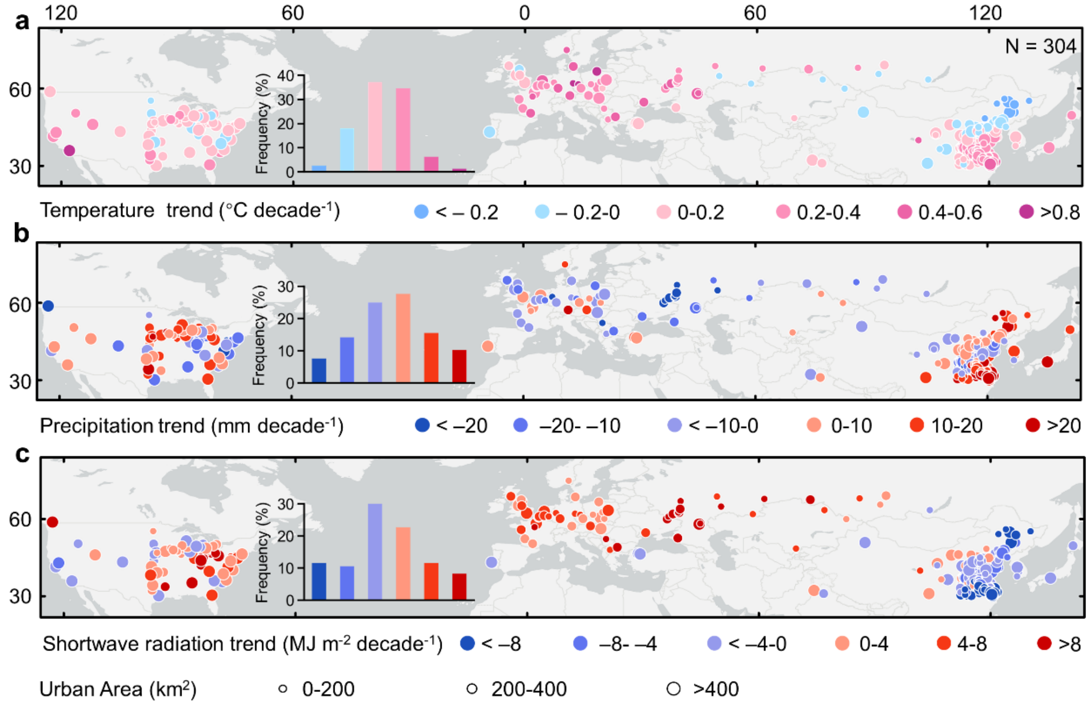


**Supplementary Figure 12.** Spatial pattern of temporal trends in the temperature (**a**), precipitation (**b**) and shortwave radiation (**c**) from 2001 to 2022. N represent the number of cities.

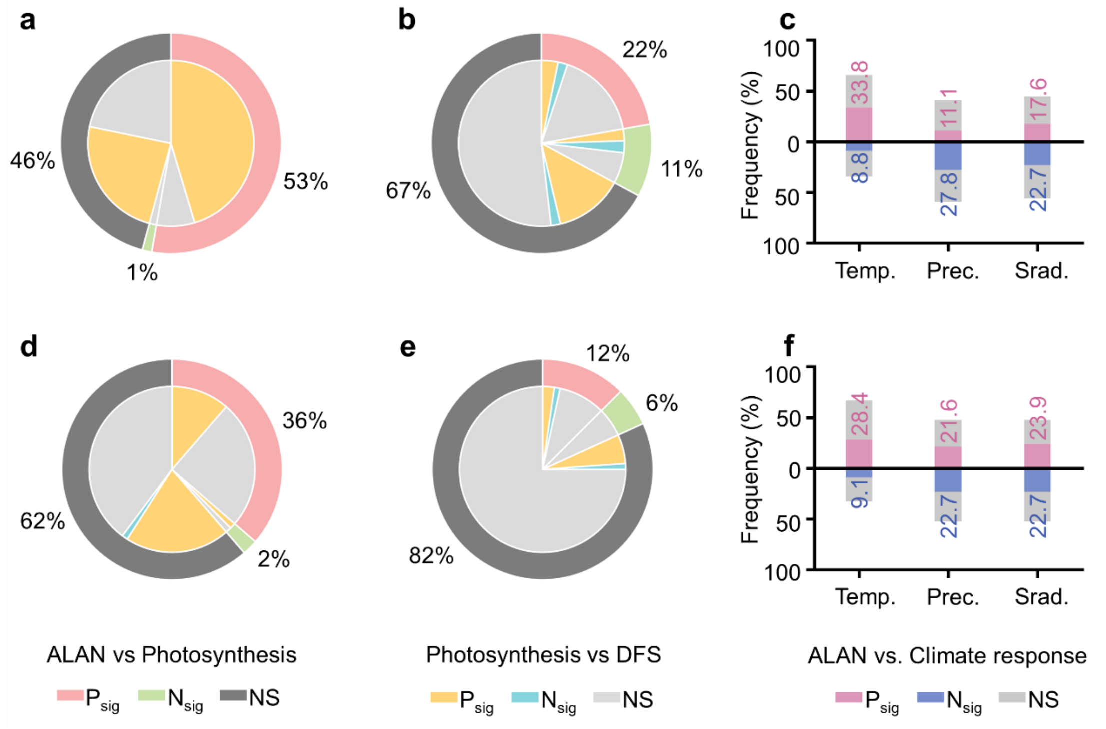


**Supplementary Figure 13.** Comparison of mediating pathways through which low- (< 15 nW $cm^{-2}$ $sr^{-1}$) and high-intensity ALAN (≥ 15 nW $cm^{-2}$ $sr^{-1}$) affect the DFS. **a, b**, Proportions of cities showing significantly positive ($P_{sig}$), negative ($N_{sig}$), and nonsignificant (NS) partial correlations between ALAN and photosynthesis (outer rings) and between photosynthesis and DFS (inner rings) under low-intensity ALAN, after controlling for temperature, precipitation, and shortwave radiation ($P < 0.05$, two-tailed *t*-test). Photosynthetic indicators include $V_{cmax}$ (**a**), SIF (**b**). **c**, Modulating effects of ALAN on climate responses under low-intensity conditions. **d**,**e**, Same as **a**, **b** but for high-intensity ALAN. **f**, Modulating effects of ALAN on climatic responses under high-intensity ALAN.

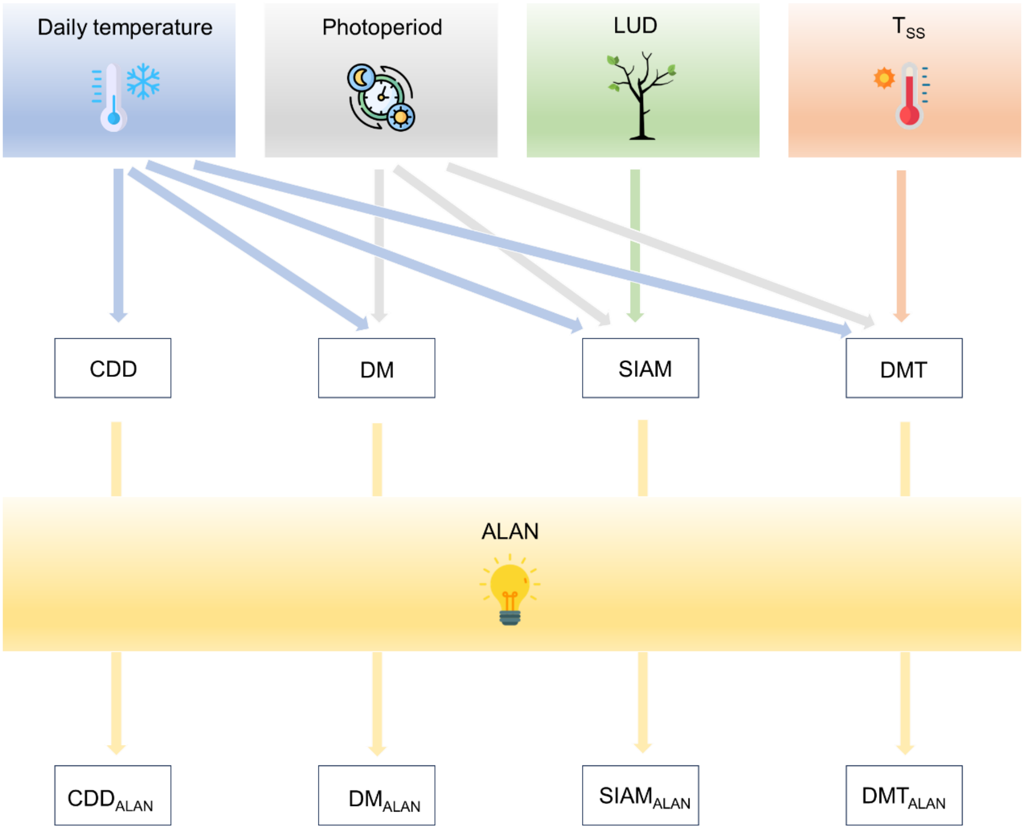


**Supplementary Figure 14.** Schematic diagram of the input parameters for four original DFS models, including the semi-empirical model based on the cooling degree days (CDD), the Delpierre model (DM), the spring-influenced autumn DFS model (SIAM), and the DM modified by spring-summer temperature (DMT), as well as their ALAN-improved versions ($CDD_{ALAN}$, $DM_{ALAN}$, $SIAM_{ALAN}$, $DMT_{ALAN}$). LUD denotes the leaf-unfolding date, and $T_{SS}$ represents the mean spring-summer temperature.

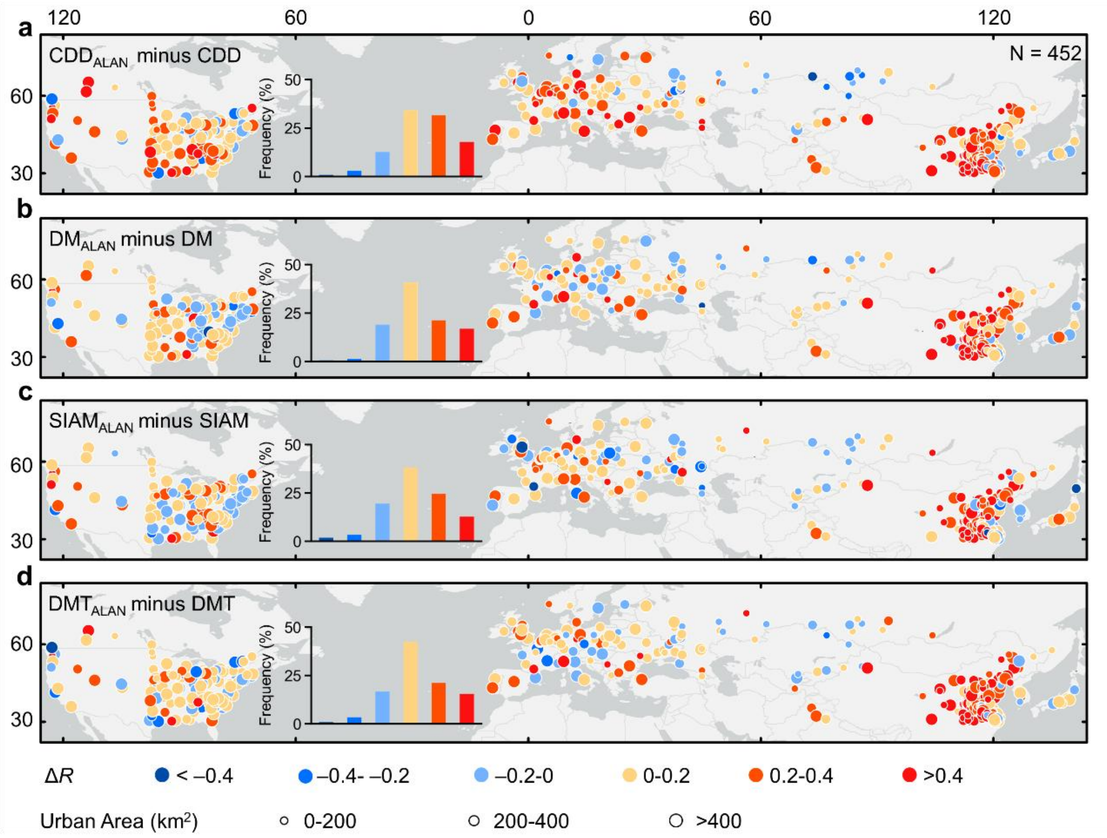


**Supplementary Figure 15.** Spatial patterns of the differences in correlation coefficients (Δ*R*) between original and improved DFS models. CDD (**a**), DM (**b**), SIAM (**c**), and DMT (**d**). N represent the number of cities.

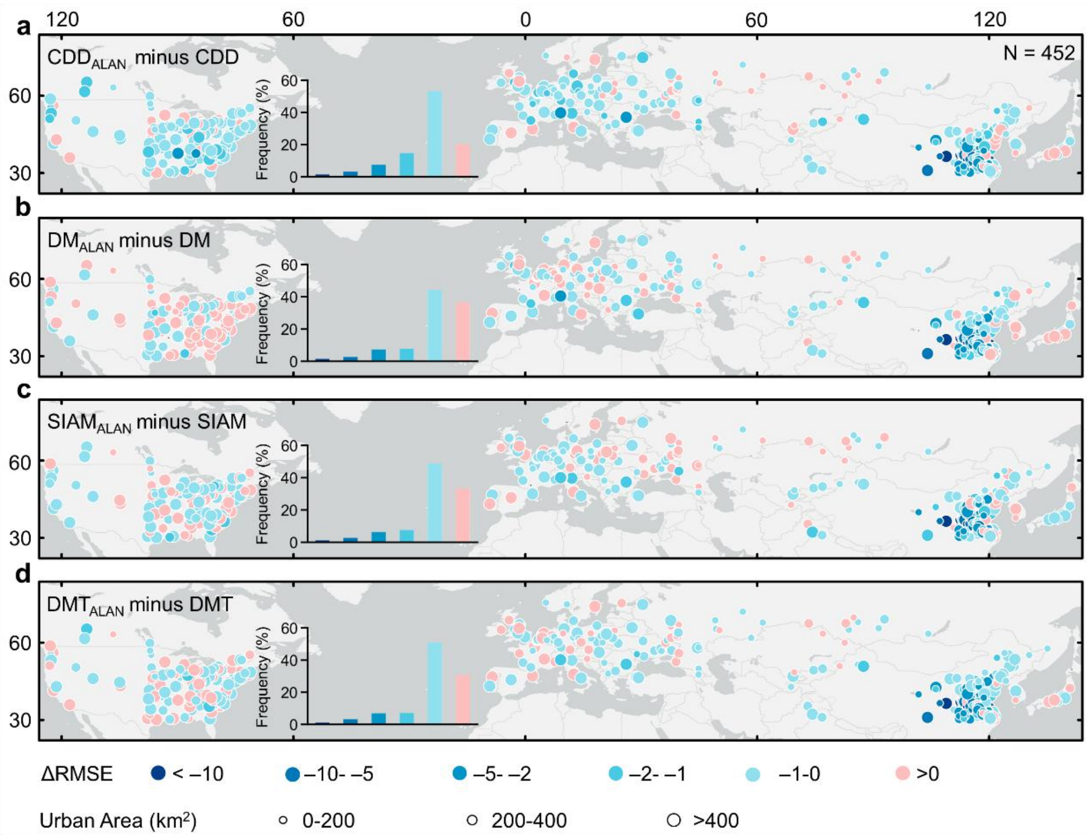


**Supplementary Figure 16.** Spatial patterns of the differences in root mean square error (ΔRMSE) between original and improved DFS models. CDD (**a**), DM (**b**), SIAM (**c**), and DMT (**d**). N represent the number of cities.

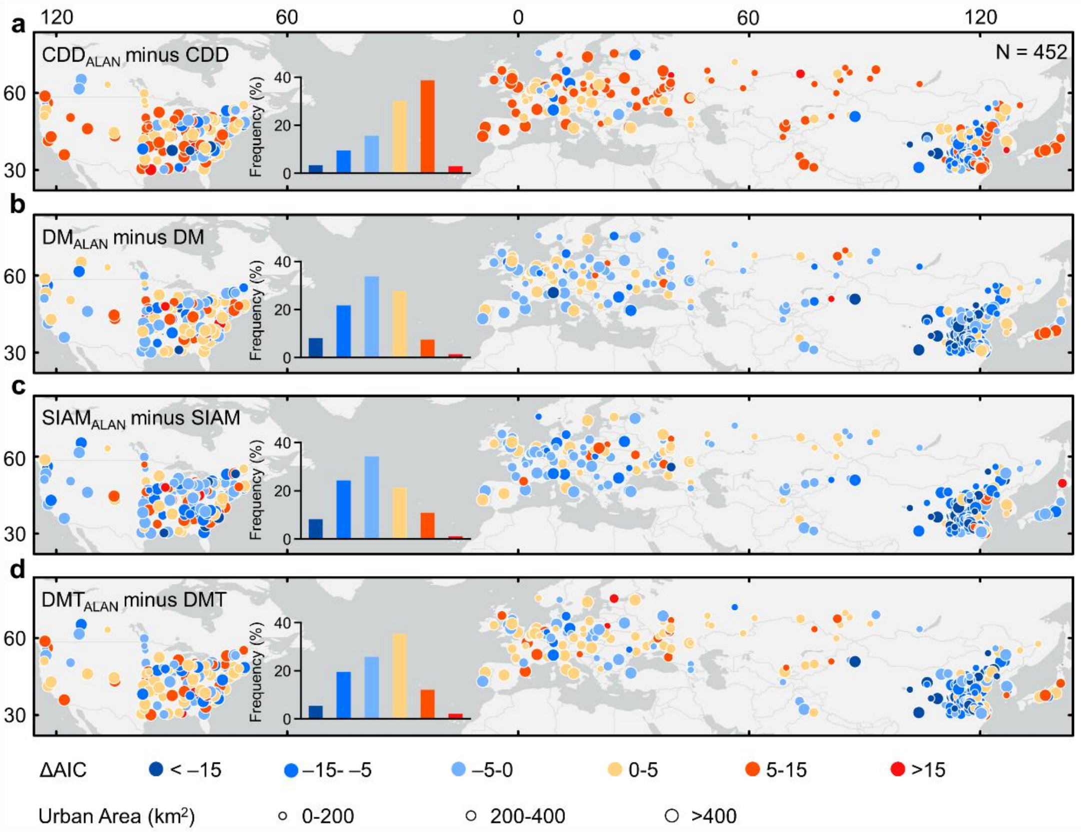


**Supplementary Figure 17.** Spatial patterns of the differences in Akaike information criterion (ΔAIC) between original and improved DFS models. CDD (**a**), DM (**b**), SIAM (**c**), and DMT (**d**). N represent the number of cities.

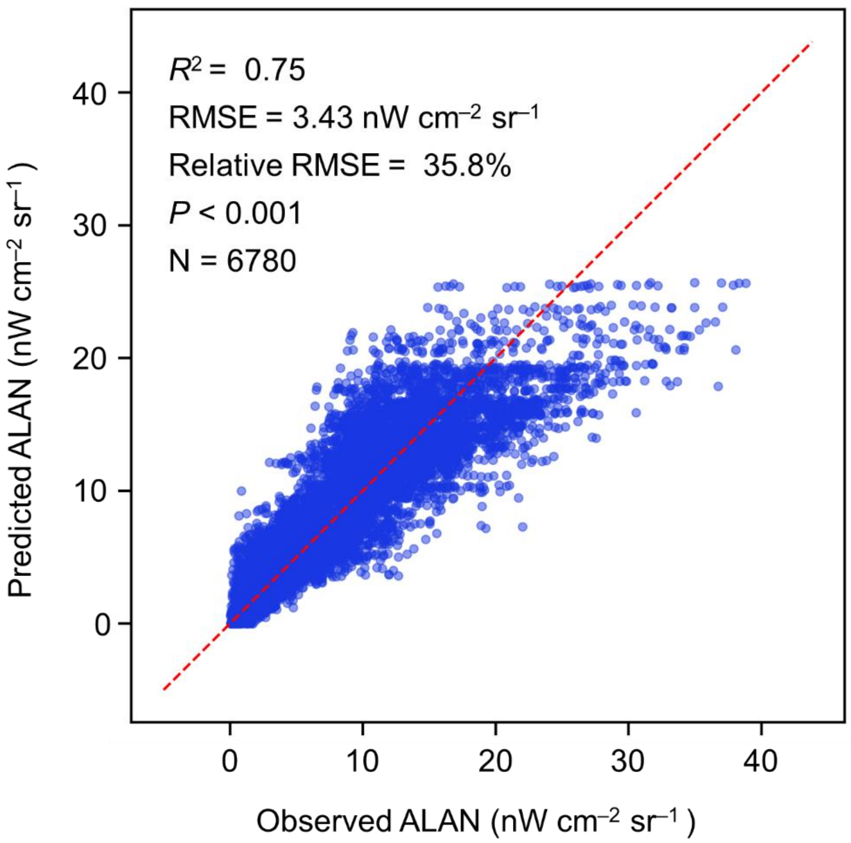


**Supplementary Figure 18.** Scatter plot of observed vs. predicted ALAN values ($P < 0.001$, two-tailed *t*-test). The diagonal line represents 1:1 reference. N represents the number of cities.

**Supplementary Table 1**. Summary of data used in this study.

| Data | Spatial resolution | Temporal resolution | Time range | Source |
|---|---|---|---|---|
| In situ DFS | In situ | yearly | 2001-2015 | PEP725[1] |
| In situ DFS | In situ | yearly | 2001-2018 | CPON[2] |
| satellite DFS, satellite GUD | 500m | yearly | 2001-2022 | MCD12Q2 v6.1[3] |
| NPP-VIIRS-like NTL | 500m | yearly | 2001-2022 | Ref[4] |
| DMSP-OLS NTL | 1km | yearly | 2001-2013 | Ref[5] |
| NPP-VIIRS NTL | 500m | monthly | 2014-2022 | Ref[6] |
| H-NTL-v2 | 1km | yearly | 2001-2022 | Ref[7] |
| global urban boundaries data | - | yearly | 2018 | Ref[8] |
| temperature | 0.5° | 6-hour | 2001-2022 | CRU JRA v2.4[9] |
| temperature, precipitation, shortwave radiation | 1/24° | monthly | 2001-2022 | TerraClimate[10] |
| HDI, GDP, Per capita GDP | 0.08333° | yearly | 2001-2015 | Ref[11] |
| aboveground biomass | 0.008333° | static | - | Ref[12] |
| tree density | 1 km | static | 2014 | Ref[13] |
| canopy height | 1km | static | - | Ref[14] |
| GPP | 500m | 8-day | 2001-2022 | MOD17A2H v006[15] |
| ET | 500m | 8-day | 2001-2022 | MOD16A2 v061[16] |
| FPAR | 500m | 8-day | 2001-2022 | MOD15A2H v061[17] |
| $V_{cmax}$ | 500m | 8-day | 2001-2019 | Vcmax25[18] |
| SIF | 0.05° | monthly | 2001-2022 | GOSIF[19] |
| future temperature | 2.5°×1.9° | daily | 2020-2100 | CMIP6 NorESM2-MM[20] |
| future Per capita GDP growth | - | 5-year | 2020-2100 | GDP[21] |

**Supplementary Table 2**. Literature support for the proposed mechanistic pathways.

| | Reference | Light type | Species type | Method | Conclusion | DOI |
|---|---|---|---|---|---|---|
| H1: Carbon sequestration | Kim et al. 2015[22] | High-pressure sodium (HPS) | Cymbidium (*Red Fire* and *Yokihi*) | Manipulative experiment. Plants in the control group grew under natural daylight conditions, while those in the treatment groups were exposed to artificial light. | Cymbidium (*Red Fire* and *Yokihi*) orchids were able to carry out photosynthesis under artificial light at night. | doi.org/10.1016/j.scienta.2015.01.036 |
| | Ermes et al.,2021[23] | Light-emitting diode (LED) | *Tilia plathyphyllos Scop.* and *Platanus x acerifolia* (Aiton) Willd | Manipulative experiment. The control group grew under natural dark conditions at night, while the experimental group was exposed to artificial light at night to simulate urban street lighting conditions. | Plants exposed to light exhibited positive nighttime photosynthetic rates, indicating active $CO_2$ assimilation compared with the control group. Moreover, chlorophyll content was higher in the plants exposed to light. | doi.org/10.12871/00021857202146 |
| | Czaja et al.,2022[24] | LED | *Cornus alba* and *Lonicera pileata* | Manipulative experiment. Three treatments were applied: control (12 h light/12 h dark), moderate light pollution (12 h light/12 h dim light), and low light pollution (12 h light/12 h intermittent light). | Light pollution treatment increased soluble sugar content in the apical twigs of *Cornus alba* and *Lonicera pileata* compared with the control. | doi.org/10.1016/j.ufug.2022.127753 |

| | | | | | | |
|---|---|---|---|---|---|---|
| | Ermes et al.2024[25] | LED | white poplar trees (*Populus alba* clone DI-1) | Manipulative experiment. The control group received no illumination after dusk, while the treatment group was exposed to street lighting at night. | Nighttime lighting allowed plants to maintain photosynthesis at night, whereas the control group primarily exhibited respiration at night. | doi.org/10.1016/j.envexpbot.2024.105861 |
| | Kolman et al. 2025[26] | LED | Common alder (*Alnus glutinosa* (L.) Gaertn.), Elder (*Sambucus nigra* L.), Filed maple (*Acer campestre* L.), Silver birch (*Betula pendula* Roth), Tree-of-heaven (*Ailanthus altissima* (Mill.) Swingle) | Manipulative experiment. Branches under streetlights and leaves from the opposite (control) side of the same trees, which received less artificial light at night, were collected for laboratory analysis. | Under strong light pollution, the fluorescence yield increased significantly in common linden and elder, and field maple, silver birch, and tree-of-heaven showed higher electron transport efficiency under street lighting. | doi.org/10.1002/pei3.70032 |
| H2: Climate responses | Bennie et al.2018[27] | LED | *A. tenuis*, *Anthoxanthum odoratum* and *H. lanatus* | Manipulative experiment. The control group vegetation was not exposed to artificial light, while the experimental groups were treated with broad-spectrum cool white LED light, and with near-monochromatic amber LED light. | When temperature cues become unreliable, plants rely more on photoperiod. Artificial light extends perceived day length, disrupting this balance and altering the strength of phenological responses to temperature variations | doi.org/10.1111/1365-2664.12927 |

| | | | | | | |
|---|---|---|---|---|---|---|
| | Meng et al. 2022[28] | NPP-VIIRS DNB (500m) | NA | Remote sensing–based study. Using remote-sensing-derived Artificial light at night (ALAN) data and phenological network observations, compared phenological values under conditions with and without ALAN. | ALAN and temperature interactively influence phenology. At lower temperatures, ALAN may amplify the autumn phenological delay caused by warming, whereas at higher temperatures, ALAN may weaken or even reverse this delay. | doi.org/10.1093/pnasnexus/pgac046 |
| | Du et al. 2022[29] | Zhao et al. 2020 (1km) | NA | Remote sensing–based study. Using remote-sensing-derived ALAN data, phenology data, and meteorological data, the differences in the effects of temperature and precipitation on autumn phenology were assessed under varying levels of ALAN. | Different levels of urbanization affect the response of the end of the growing season (EOS) to changes in temperature and precipitation.(with urbanization level represented by nighttime light intensity). | doi.org/10.1016/j.jag.2022.103086 doi.org/10.1109/TGRS.2019.2949797 |
| | Wang et al. 2025[30] | NPP-VIIRS DNB (500m) | NA | Remote sensing–based study. Using remote-sensing-derived phenology data, ALAN data, and temperature data, partial correlation analysis was conducted to assess the effects of ALAN and temperature on phenology. | ALAN played a more important role in delaying EOS than air temperature, but its effect diminished as city temperatures increased. | doi.org/10.1038/s44284-025-00258-2 |

**References**


1. Templ, B. et al. Pan European Phenological database (PEP725): a single point of access for European data. *Int. J. Biometeorol.* **62**, 1109–1113 (2018).
2. Ge, Q., Wang, H., Rutishauser, T. & Dai, J. Phenological response to climate change in China: a meta-analysis. *Glob. Chang. Biol.* **21**, 265–274 (2015).
3. Gray, J., Sulla-Menashe, D. & Friedl, M. A. User Guide to Collection 6 MODIS Land Cover Dynamics (MCD12Q2) Product (NASA EOSDIS LP DAAC, 2019).
4. Chen, Z. et al. An extended time series (2000–2018) of global NPP-VIIRS-like nighttime light data from a cross-sensor calibration. *Earth Syst. Sci. Data* **13**, 889–906 (2021).
5. Li, X., Zhou, Y., Zhao, M., & Zhao, X. A harmonized global nighttime light dataset 1992–2018. *Sci. Data* **7**, 168 (2020).
6. Elvidge, C. D., Baugh, K., Zhizhin, M., Hsu, F. C. & Ghosh, T. VIIRS night-time lights. *Int. J. Remote Sens*. **38**, 5860–5879 (2017).
7. Geng, M. et al. An efficient method for aurora and noise reduction with a harmonized nighttime light dataset. *Remote Sens. Environ.* **328**, 114891 (2025).
8. Gong, P. et al. Annual maps of global artificial impervious area (GAIA) between 1985 and 2018. *Remote Sens. Environ.* **236**, 111510 (2020).
9. Harris, I., Osborn, T. J., Jones, P. & Lister, D. Version 4 of the CRU TS monthly high-resolution gridded multivariate climate dataset. *Sci. Data.* **7**, 109 (2020).
10. Abatzoglou, J. T., Dobrowski, S. Z., Parks, S. A. & Hegewisch, K. C. TerraClimate, a high-resolution global dataset of monthly climate and climatic water balance from 1958–2015. *Sci. Data.* **5**, 1–12 (2018).
11. Kummu, M., Taka, M. & Guillaume, J. H. A. Gridded global datasets for Gross Domestic Product and Human Development Index over 1990–2015. *Sci. Data.* **5**, 180004 (2018).
12. Mo, L. et al. The global distribution and drivers of wood density and their impact on forest carbon stocks. *Nat. Ecol. Evol.* **8**, 2195–2212 (2024).
13. Crowther, T. W. et al. Mapping tree density at a global scale. *Nature* **525**, 201–205 (2015).
14. Simard, M., Pinto, N., Fisher, J. B. & Baccini, A. Mapping forest canopy height globally

with spaceborne lidar. *J. Geophys. Res.* **116**, G04021 (2011).

15. Running, S. W. et al. A Continuous Satellite-Derived Measure of Global Terrestrial Primary Production. *BioScience* **54**, 547–560 (2004).
16. Mu, Q., Zhao, M. & Running, S. W. Improvements to a MODIS global terrestrial evapotranspiration algorithm. *Remote Sens. Environ.* **115**, 1781–1800 (2011).
17. Myneni, R. B. et al. Global products of vegetation leaf area and fraction absorbed PAR from year one of MODIS data. *Remote Sens. Environ.* **83**, 214–231 (2002).
18. Lu, X. et al. Maximum carboxylation rate estimation with Chlorophyll content as a proxy of Rubisco content. *J. Geophys. Res. Biogeosci.* **125**, e2020JG005748 (2020).
19. Li, X. & Xiao, J. A Global, 0.05-Degree Product of Solar-Induced Chlorophyll Fluorescence Derived from OCO-2, MODIS, and Reanalysis Data. *Remote Sens.* **11**, 517 (2019).
20. Seland, Ø. et al. Overview of the Norwegian Earth System Model (NorESM2) and key climate response of CMIP6 DECK, historical, and scenario simulations. *Geosci. Model Dev.* **13**, 6165–6200 (2020).
21. Riahi, K. et al. The Shared Socioeconomic Pathways and their energy, land use, and greenhouse gas emissions implications: An overview. *Glob. Environ. Chang.* **42**, 153–168 (2017).
22. Kim, Y. J. et al. Photosynthetic changes in Cymbidium orchids grown under different intensities of night interruption lighting. *Sci. Hortic.* **186**, 124-128 (2015).
23. Lo Piccolo, E., Lauria, G. & Remorini, D. Urban lighting alters chlorophyll metabolism and romotes $CO_2$ assimilation during the night in Tilia plathyphyllos Scop. and Platanus x acerifolia (Aiton) Willd. *Agrochimica* **65**, 389-400 (2021).
24. Czaja, M. & Kołton, A. How light pollution can affect spring development of urban trees and shrubs. *Urban For. Urban Green.* **77**, 127753 (2022).
25. Piccolo, E. L. et al. LED streetlamps alter tree architecture, downregulate the photosynthetic process and alter the sugar metabolism of Populus alba L. *Environ. Exp. Bot.* 226, 105861 (2024).
26. Kolman, F., Kolláth, Z., Molnár, P. & Skribanek, A. The Effect of 3000 K LED Lamps on the Photosynthesis and Morphology of Deciduous Tree Species. *Plant. Environ.*

*Interact*. **6**, e70032 (2025).

27. Bennie, J., Davies, T. W., Cruse, D., Bell, F. & Gaston, K. J. Artificial light at night alters grassland vegetation species composition and phenology. *J. Appl. Ecol.* **55**, 442–450 (2018).
28. Meng, L. et al. Artificial light at night: an underappreciated effect on phenology of deciduous woody plants. *PNAS Nexus* **1**, pgac046 (2022).
29. Du, H. et al. Responses of autumn vegetation phenology to climate change and urbanization at northern middle and high latitudes. *Int. J. Appl. Earth Obs. Geoinf.* **115**, 103086 (2022).
30. Wang, L. et al. Artificial light at night outweighs temperature in lengthening urban growing seasons. *Nat. Cities* **2**, 506–517 (2025).
