# Supplementary material for: Increased artificial illumination delays urban autumnal foliar senescence
Source: Nat Commun. 2026 Jan 3;17:1526. doi: 10.1038/s41467-025-68246-7 (PMC12891584; doi:10.1038/s41467-025-68246-7)
Supplement: Supplementary file 3 — Reporting Summary [file 41467_2025_68246_MOESM3_ESM.pdf]

nature portfolio

Corresponding author(s): Chaoyang Wu
Last updated by author(s): Dec 16, 2025

Reporting Summary

Nature Portfolio wishes to improve the reproducibility of the work that we publish. This form provides structure for consistency and transparency in reporting. For further information on Nature Portfolio policies, see our Editorial Policies and the Editorial Policy Checklist.

Statistics

For all statistical analyses, confirm that the following items are present in the figure legend, table legend, main text, or Methods section.

n/a	Confirmed	
☐	☒	The exact sample size (n) for each experimental group/condition, given as a discrete number and unit of measurement
☒	☐	A statement on whether measurements were taken from distinct samples or whether the same sample was measured repeatedly
☐	☒	The statistical test(s) used AND whether they are one- or two-sided<br>*Only common tests should be described solely by name; describe more complex techniques in the Methods section.*
☐	☒	A description of all covariates tested
☒	☐	A description of any assumptions or corrections, such as tests of normality and adjustment for multiple comparisons
☐	☒	A full description of the statistical parameters including central tendency (e.g. means) or other basic estimates (e.g. regression coefficient) AND variation (e.g. standard deviation) or associated estimates of uncertainty (e.g. confidence intervals)
☐	☒	For null hypothesis testing, the test statistic (e.g. F, t, r) with confidence intervals, effect sizes, degrees of freedom and P value noted<br>*Give P values as exact values whenever suitable.*
☒	☐	For Bayesian analysis, information on the choice of priors and Markov chain Monte Carlo settings
☒	☐	For hierarchical and complex designs, identification of the appropriate level for tests and full reporting of outcomes
☒	☐	Estimates of effect sizes (e.g. Cohen's d, Pearson's r), indicating how they were calculated

Our web collection on statistics for biologists contains articles on many of the points above.

Software and code

Policy information about availability of computer code

Data collection	The data used in this study is public available, no software and code is needed for data collection.
Data analysis	All data analyses and modeling were performed using Python (v3.8.10). The code is stored in a publicly available Zenodo repository https://doi.org/10.5281/zenodo.17925641.

For manuscripts utilizing custom algorithms or software that are central to the research but not yet described in published literature, software must be made available to editors and reviewers. We strongly encourage code deposition in a community repository (e.g. GitHub). See the Nature Portfolio guidelines for submitting code & software for further information.

Data

Policy information about availability of data

All manuscripts must include a data availability statement. This statement should provide the following information, where applicable:

- Accession codes, unique identifiers, or web links for publicly available datasets
- A description of any restrictions on data availability
- For clinical datasets or third party data, please ensure that the statement adheres to our policy

All data used in this study are freely available from the following sources: In situ DFS data can be accessed from https://doi.org/10.5281/zenodo.17925641 and http://www.pep725.eu/ . Satellite-derived DFS data is available from https://lpdaac.usgs.gov/products/mcd12q2v061/. NPP-VIIRS-like nighttime light data is available from https://dataverse.harvard.edu/dataset.xhtml?persistentId=doi:10.7910/DVN/YGIVCD. DMSP-OLS nighttime light data is available from https://eogdata.mines.edu/products/dmsp/. NPP-VIIRS nighttime light data is available from https://eogdata.mines.edu/products/vnl/. H-NTL-v2 data is available from

https://doi.org/10.5281/zenodo.17925641. Global urban boundaries data is available from https://data-starcloud.pcl.ac.cn/iearthdata/map?id=14. Six-hourly temperature data is available from https://catalogue.ceda.ac.uk/uuid/aed8e269513f446fb1b5d2512bb387ad/. Monthly climatic data is available from https://www.climatologylab.org/terraclimate.html. HDI, GDP, Per capita GDP data is available from https://datadryad.org/dataset/doi:10.5061/dryad.dk1j0. Aboveground biomass is available from https://zenodo.org/records/13331493. Tree density is available from https://elischolar.library.yale.edu/yale_fes_data/1/. Canopy height is available from https://webmap.ornl.gov/ogc/dataset.jsp?ds_id=1665. GPP, ET, FPAR data are available from https://lpdaac.usgs.gov/products/. Vcmax data is available from https://www.nesdc.org.cn/sdo/detail?id=612f42ee7e28172cbed3d80f. SIF is available from https://globalecology.unh.edu/data/GOSIF.html. Future temperatures, Per capita GPP data were from the CMIP6 models (https://esgf-node.llnl.gov/projects/esgf-llnl/). Source data are provided with this paper.

Research involving human participants, their data, or biological material

Policy information about studies with human participants or human data. See also policy information about sex, gender (identity/presentation), and sexual orientation and race, ethnicity and racism.

Reporting on sex and gender	N/A
Reporting on race, ethnicity, or other socially relevant groupings	N/A
Population characteristics	N/A
Recruitment	N/A
Ethics oversight	N/A

Note that full information on the approval of the study protocol must also be provided in the manuscript.

Field-specific reporting

Please select the one below that is the best fit for your research. If you are not sure, read the appropriate sections before making your selection.

☐ Life sciences ☐ Behavioural & social sciences ☒ Ecological, evolutionary & environmental sciences

For a reference copy of the document with all sections, see nature.com/documents/nr-reporting-summary-flat.pdf

Ecological, evolutionary & environmental sciences study design

All studies must disclose on these points even when the disclosure is negative.

Study description	This study investigated the effect of artificial light at night (ALAN) on the dates of foliar senescence (DFS) in urban areas.
Research sample	62,994 site-year records of DFS<br>satellite observations of ALAN and DFS across 452 cities from 2001 to 2022
Sampling strategy	The analyzed data in our study were obtained from open-access database instead of designed experiments, sampling is not applicable to our study.
Data collection	All data used in this study are available online. The specific links for each dataset are presented in Data availability.
Timing and spatial scale	Between 2001-2022.<br>Urban areas for temperate and high latitudes (> 30°N)
Data exclusions	For ground observations of leaf green-up dates, we applied the median absolute deviation (MAD) method to identify and remove outliers. We also excluded all ground records that were shorter than 10 years to make temporal analyses more reliable.
Reproducibility	The phenology, PM2.5 data, climate data and models are open access. The findings of our study can be reproduced using the statistical methods shown in the manuscript.
Randomization	The analyzed data in our study were obtained from open-access database instead of designed experiments, randomization is not applicable to our study.
Blinding	The analyzed data in our study were obtained from open-access database instead of designed experiments, blinding is not applicable to our study.

Did the study involve field work? ☐ Yes ☒ No

Reporting for specific materials, systems and methods

We require information from authors about some types of materials, experimental systems and methods used in many studies. Here, indicate whether each material, system or method listed is relevant to your study. If you are not sure if a list item applies to your research, read the appropriate section before selecting a response.

Materials & experimental systems

n/a	Involved in the study
☒	☐ Antibodies
☒	☐ Eukaryotic cell lines
☒	☐ Palaeontology and archaeology
☒	☐ Animals and other organisms
☒	☐ Clinical data
☒	☐ Dual use research of concern
☒	☐ Plants

Methods

n/a	Involved in the study
☒	☐ ChIP-seq
☒	☐ Flow cytometry
☒	☐ MRI-based neuroimaging

Plants

Seed stocks	N/A
Novel plant genotypes	N/A
Authentication	N/A
